# Informed consent to midwifery practices and interventions during the second stage of labor—An observational study within the Oneplus trial

**DOI:** 10.1371/journal.pone.0304418

**Published:** 2024-06-12

**Authors:** Cecilia Häggsgård, Christine Rubertsson, Pia Teleman, Malin Edqvist

**Affiliations:** 1 Department of Health Sciences, Medical Faculty, Lund University, Lund, Sweden; 2 Department of Obstetrics and Gynecology, Skane University Hospital, Lund, Sweden; 3 Department of Clinical Sciences, Lund University, Lund, Sweden; 4 Clinical Epidemiology Unit, Department of Medicine Solna, Karolinska Institutet, Stockholm, Sweden; 5 Department of Women´s Health and Health Professions, Karolinska University Hospital, Stockholm, Sweden; University of Gothenburg: Goteborgs Universitet, SWEDEN

## Abstract

**Objectives:**

To study informed consent to midwifery practices and interventions during the second stage of labor and to investigate the association between informed consent and experiences of these practices and interventions and women’s experiences of the second stage of labor.

**Methods:**

This study uses an observational design with data from a follow-up questionnaire sent to women one month after giving birth spontaneously in the Oneplus trial, a study aimed at evaluating collegial midwifery assistance to reduce severe perineal trauma. The trial was conducted between 2018–2020 at five Swedish maternity wards and trial registered at clinicaltrials.gov, no NCT03770962. The follow-up questionnaire contained questions about experiences of the second stage of labor, practices and interventions used and whether the women had provided informed consent. Evaluated practices and interventions were the use of warm compresses held at the perineum, manual perineal protection, vaginal examinations, perineal massage, levator pressure, intermittent catheterization of the bladder, fundal pressure, and episiotomy. Associations between informed consent and women’s experiences were assessed by univariate and multivariable logistic regression.

**Findings:**

Of the 3049 women participating in the trial, 2849 consented to receive the questionnaire. Informed consent was reported by less than one in five women and was associated with feelings of being safe, strong, and in control. Informed consent was further associated with more positive experiences of clinical practices and interventions, and with less discomfort and pain from interventions involving physical penetration of the genital area.

**Conclusion:**

The findings indicate that informed consent during the second stage is associated with feelings of safety and of being in control. With less than one in five women reporting informed consent to all practices and interventions performed by midwives, the results emphasize the need for further action to enhance midwives’ knowledge and motivation in obtaining informed consent prior to performance of interventions.

## Introduction

The second stage of labor has been referred to as the momentous culmination of childbirth with increased emotional and physical intensity for the woman giving birth [1, 2]. Midwifery care during this stage aims to optimize women’s birth experiences and secure the wellbeing of the woman and the baby [3, 4]. To achieve this, midwives work in partnership with women, provide continuous support and focus on each woman’s individual needs and preferences [4, 5]. During the second stage of labor midwives use a variety of practices and interventions to facilitate labor, accelerate the birth process if needed, and act to prevent perineal trauma [6–9]. Obstetric interventions are defined as therapeutic measures taken to safeguard or improve the health of the pregnant woman and the fetus [10]. For the second stage of labor this includes vaginal examinations, intermittent catheterization of the bladder, levator pressure, i.e. digital stretching of the vagina, fundal pressure and episiotomy [3, 9, 11]. Warm compresses held at the perineum, manual perineal support and massage of the perineum have been defined as second stage midwifery practices [12]. Previous research shows that interventions used during the second stage are advantageous when required, but potentially harmful if used inappropriately, and it is also found that such procedures can cause morbidity, distress and pain [13–16]. The use of interventions during the second stage of labor has been associated with negative birth experiences and dissatisfaction with care in several studies [3, 17–19]. Interventions assessed to have adverse effects or with the potential to be harmful are fundal pressure [3, 17], episiotomy [18] and vaginal examinations [19]. Vaginal examinations and massage of the perineum have furthermore been associated with discomfort and feelings of being vulnerable and exposed [3, 20].

The lack of informed consent and disregard for women’s autonomy during childbirth is a globally recognized issue [21]. Being involved in the decision-making process throughout labor and birth has been demonstrated to exert a more significant impact on women’s childbirth experiences compared to other factors such as labor pain and medical interventions [22–24]. Informed consent involves a process of information exchange about a medical procedure between the health care provider and the patient, followed by an intentional and voluntary decision from the patient [25, 26]. Available alternatives should be presented to the pateint, including the option to decline [25]. However, health care necessary to avoid harm that seriously threatens the life or health of the patient should be provided even if he or she for any reason cannot consent [26]. Lack of informed consent has been defined as a form of abuse which includes disrespectful and neglectful care or abusive treatment [27]. Procedures performed without consent violate fundamental legal rights of women in many countries, including Sweden where it is regulated in the patient act [26]. According to the Swedish patient act the patient can provide written or oral informed consent or by other means demonstrate that he or she consent to the proposed intervention. Lack of participation and disrespectful care has been associated with negative or traumatic birth experiences in several studies [16, 20, 28]. A Dutch study on women’s experiences of vaginal examinations revealed that 35% had negative experiences of the procedure, with the odds increasing based on the number of examinations performed [19]. Due to this evidence, there has been a notable increase in attention during the past decade paid to childbirth situations characterized by lack of informed consent and a disregard for women’s autonomy [18, 27, 29–31].

There is limited understanding on how midwifery practices, interventions and informed consent during the second stage of labor can impact women’s experiences during this specific stage [9, 12].

The Oneplus trial was designed to evaluate the effect of an intervention including two midwives assisting women during the late second stage of labor with the purpose to reduce severe perineal trauma [32]. Within the project, women’s experiences of the second stage of labor and midwifery practices employed during this stage were also assessed. Therefore, the objective was to study informed consent to midwifery practices and interventions during the second stage of labor and to investigate the association between informed consent to these practices and interventions and women’s experiences of the second stage of labor.

## Methods

### Study design

This study uses an observational design with data from a follow-up questionnaire sent to women one month after birth and who participated in the Oneplus trial [32]. The Oneplus trial is a multicenter randomized controlled trial conducted to test the hypothesis that the presence of a second midwife during the second stage of labor, with the purpose of preventing severe perineal trauma, would result in fewer injuries affecting the anal sphincter than if attended by one midwife. Data was collected between December 2018 and May 2020. The results from the Oneplus trial with further details have been reported elsewhere [32].

### Study context

In Sweden, midwives are the primary caregivers during pregnancy, labor and birth. The model of care is fragmented, meaning that different care providers are responsible for the care of the woman from pregnancy up until the postnatal period, and that a change of caregiver may occur during labor, due to shift change.

### Participants

Inclusion criteria in the Oneplus trial were: 18–47 years of age, opting for giving birth vaginally for the first time, either primiparous or with one previous caesarean section, at least 37+0 gestational weeks, and with a singleton live fetus in the vertex presentation. To provide written informed consent, the women needed to be proficient in Swedish, English, Arabic, or Farsi. To be able to participate in the follow-up questionnaire, the women needed to have mastered the Swedish or English language. Women were recruited when admitted to the obstetric unit for labor and birth. In total, 3059 women gave birth spontaneously and were attended by one or by two midwives in the trial. Of those women, 2831 (92.5%) consented to receive a follow-up questionnaire one month after the birth.

### Questionnaire sent to women one month after birth

The follow-up questionnaire sent to women one month after birth was developed before the onset of the trial to assess short-term secondary outcomes including experiences of the second stage of labor. Since no validated scales on the subject were found, and since existing scales focus on the overall birth experience [33], study-specific items were developed. Items regarding experiences of the second stage of labor were constructed based on the Childbirth Experience Questionnaire (CEQ) [34] research findings [1], literature [4] and clinical experience. Midwifery practices that were of interest included the use of warm compresses held at the perineum, manual perineal protection and perineal massage [12]. Vaginal examinations, levator pressure, intermittent catheterization of the bladder, fundal pressure and episiotomy were defined as interventions. Since these practices and interventions could be experienced as both harmful and helpful, positive as well as negative response options were included.

To assess the face validity of the items, cognitive interviewing [35] was conducted with ten women who had recently given birth spontaneously. A think-aloud approach was used during the interviews [36]. Amendments proposed by the women were discussed in the research group in order to retain the meaning of the items. Adjustments were made to the wording of some items as proposed by the women to make the items acceptable and comprehensive. When tested in the next step, the women offered no additional comments on the items.

The questionnaires were provided electronically to women who had provided email addresses, or as postal questionnaires if they had not. The English version of the questionnaire was only available as a postal questionnaire. Four reminders were sent out to increase participation.

### Data collection

In addition to data from the follow-up questionnaire, this study also uses data from the Oneplus trial. Trial data included ethnicity, pre-pregnancy body mass index (BMI), parity, onset of labor, epidural analgesia, augmentation with oxytocin, duration of the total and the active second stage of labor and birth position. The following continuous variables were categorized: maternal age into <25 years, 25–35 years, >35 years and BMI according to the WHO classification (<18.5, 18.5–24.9, 25.0–30.0, >30.0).

The follow-up questionnaire included questions regarding sociodemographic variables, i.e., level of education, marital status, and mental ill-health before pregnancy. The follow-up questionnaire included 15 items regarding experiences of the second stage of labor (S1 Appendix). For the purpose of this study eight items regarding experiences of the second stage of labor (Box 1) and eight items regarding midwifery practices and interventions were used (Box 2). Six of the items regarding the experience of the second stage of labor were rated on a 4-point Likert scale ranging from 1 (Strongly agree) to 4 (Disagree), and two items were rated on a 7-point scale ranging from 1 (Not in control) to 7 (Completely in control), and 1 (Very unsafe) to 7 (Totally safe) (Box 1). The selection of variables was based on previous literature and discussions within the research group and was designed to capture the relevant aspects of the experience of the second stage of labor and informed consent.

**Box 1. Items regarding experiences of the second stage of labor**

**Items rated on 4-point Likert scales**

I felt strong during the second stage of labor*

I felt vulnerable during the second stage of labor^†^

I have positive memories from the second stage of labor*

I have negative memories from the second stage of labor^†^

The midwife understood my needs during the second stage of labor*

I felt that I could handle the situation during the second stage of labor*

**Items rated on 7-point Likert scales**

How much of a feeling of being in control did you experience during the second stage of labor? *Not in control (1)—Completely in control (7)*^‡^

When you look back on the birth now, how safe did you feel during the second stage of labor? *Very unsafe (1)—Totally safe (7)*^‡^

*Dichotomized (strongly agree/mostly agree, agree in part, disagree)

^†^Dichotomized (strongly agree, mostly agree, agree in part/disagree)

^‡^Dichotomized (1-5/6-7)

**Box 2. Items regarding the use of midwifery practices and interventions during the second stage of labor**

The midwife used warm compresses during the second stage of labor

The midwife applied perineal support with her hands and supported the baby’s head during the second stage of labor

The midwife massaged my perineum with oil

The midwife performed vaginal examinations several times during the second stage of labor

The midwife showed me how to push by putting her fingers in my vagina and pressing down during the second stage of labor

The midwife used catheterization to empty my bladder during the second stage of labor

The midwife pressed down on my uterus to assist in shortening the second stage of labor

The midwife performed an episiotomy (cut to the perineum with scissors)

**Each of the statements above was followed by another five statements rated on 4-point Likert scales ranging from 1 (Strongly agree) to 4 (Disagree)**:

The experience was positive*

It was unpleasant^†^

It was painful^†^

I received information about the procedure*

I was given the option to decline the procedure*

*Dichotomized (strongly agree/mostly agree, agree in part, disagree)

^†^Dichotomized (strongly agree, mostly agree, agree in part/disagree)

For each practice and intervention there were three response options; “yes”, “no” and “cannot recall”. Women who recalled the use of a midwifery practice or intervention were further asked to answer five statements regarding experiences of the practice or intervention, assessed on a four-point Likert scale, ranging from 1 (Strongly agree) to 4 (Disagree). These statements included whether the women experienced the practice or intervention as positive, unpleasant or painful, whether they had received information about the procedure, and whether they felt that they had the option to decline (Box 2). The latter two statements were created as a proxy of informed consent based on existing definitions [25, 26].

#### Outcome variables

To study the association between informed consent to midwifery practices and interventions and women´s experiences two outcome variables were used: *The experience was positive* and *It was painful and/or unpleasant*. The latter variable was created of the two statements *It was painful* and *It was unpleasant*. All Likert-scale responses were dichotomized so that the most positive statement on a Likert-scale could be compared with all other statements [37]. For the variable *The experience was positive* this meant that women who answered “Strongly agree” were compared with those who answered “Mostly agree”, “Agree in part” and “Disagree”. For the variable *It was painful and/or unpleasant* women who responded, “Strongly agree”, “Mostly agree” or “Agree in part” to one or both statements were compared with women who responded “Disagree” on both statements.

In addition, the eight variables regarding women’s experiences during the second stage of labor were used as outcome variables. The following first six items involved rating on a 4-point Likert scale: *I felt strong during the second stage of labor*, *I felt vulnerable during the second stage of labor*, *I have positive memories from the second stage of labor*, *I have negative memories from the second stage of labor*, *The midwife understood my needs during the second stage of labor* and *I felt that I could handle the situation during the second stage of labor*. Responses for positively worded items were dichotomized as “Strongly agree” compared with “Mostly agree”, “Agree in part”, and “Disagree”, whereas negatively worded items were dichotomized as “Strongly agree”, “Mostly agree” and “Agree in part” compared with “Disagree”. The two subsequent items involved rating on a 7-point scale: *How much of a feeling of being in control did you experience during the second stage of labor*? and *When you look back on the birth now*, *how safe did you feel during the second stage of labor*?. Responses were dichotomized as 1–5 compared with 6–7 (Box 1).

#### Explanatory variables

**Fig 1:**
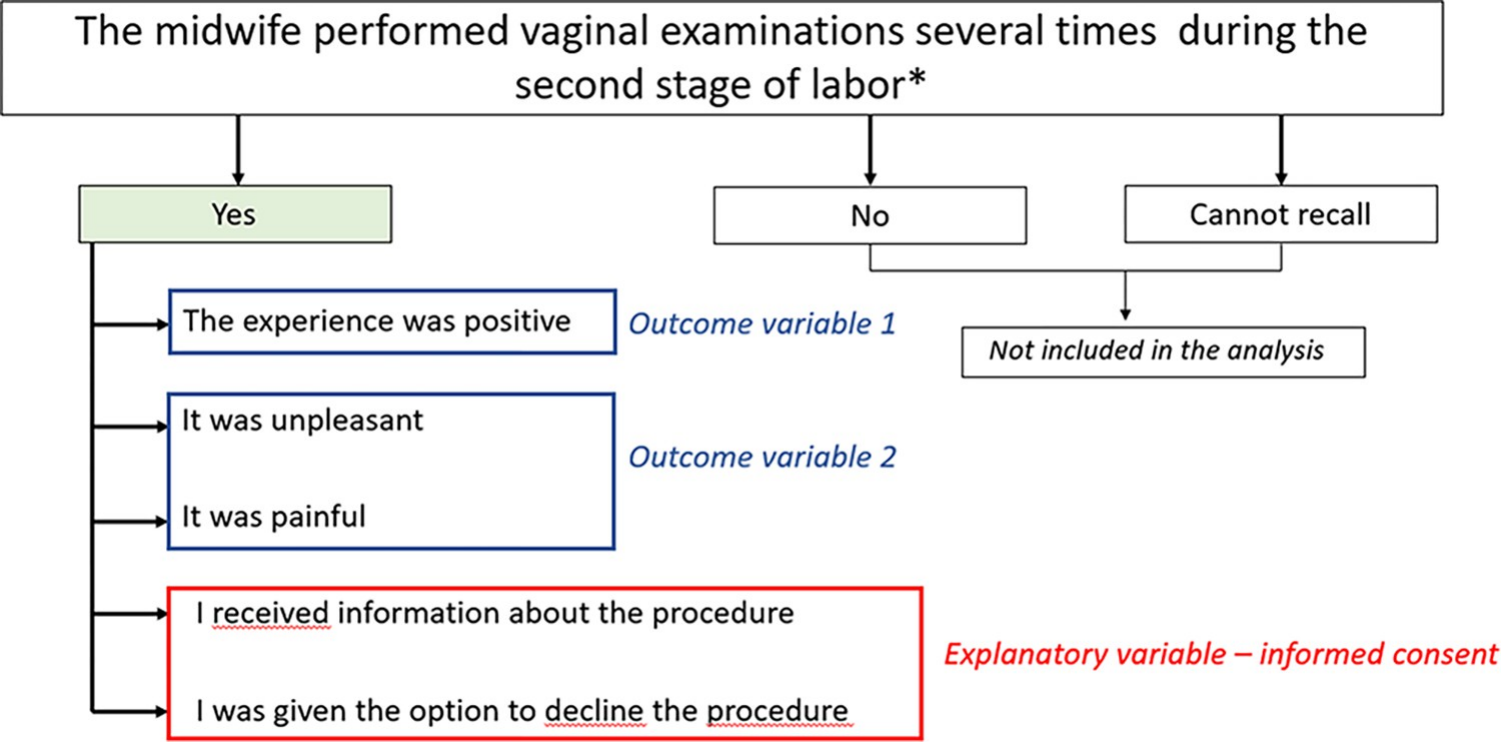
Representation of outcome variables and the explanatory variable. *Example of a question regarding midwifery practice/intervention used during the second stage of labor.

To be able to capture the concept of informed consent, a combined variable was designed on the basis of responses to the two statements; *I received information about the procedure*, and *I was given the option to decline the procedure*. The combined variable is used as a proxy for informed consent. This decision was based on existing definitions [25, 26], where the key principle of Swedish law stipulates that informed consent include receiving information and the possibility to decline. Informed consent was considered fulfilled if the women had responded “Strongly agree” on both statements. Women who stated that they had been exposed to any or all of the eight midwifery practices or interventions were divided into two groups: *Informed consent* and *No informed consent*. The *Informed consent* group was formed by those who had provided informed consent to all midwifery practices and interventions they had experienced. The *No informed consent* group included women who for any of the practices or interventions responded “Mostly agree”, “Agree in part” or “Disagree” on one or both of the statements *I received information about the procedure* and *I was given the option to decline the procedure* (Fig 1).

#### Covariates

Clinical reasoning and the use of Directed Acyclic Graphs [38] were used to identify potential confounders. The chosen potential confounders were: Swedish as native language, fear of birth, level of education and length of the second stage. As two of the interventions, fundal pressure and episiotomy, contained few observations [39], the regression model only included level of education and fear of birth as confounders for fundal pressure and level of education, fear of birth and Swedish as native language as confounders for episiotomy.

### Statistical analyses

Descriptive statistics were used to present the study sample. Chi square tests, Student’s *t* tests and Mann Whitney U tests were used to compare the two groups *Informed consent* and *No informed consent* depending on the quality of the variables. The level of significance was set to <0.05 for all analyses. To study women’s experiences between the two groups (*Informed consent vs No informed consent*), univariate and multivariable logistic regression modeling was performed to calculate crude and adjusted odds ratios with a 95% confidence interval (CI). The analyses were conducted using IBM SPSS software (version 28).

### Ethical approval

Ethical approval for the trial was obtained from the Regional Ethics Committee in Lund, Sweden (number 2018–476).

## Findings

**Fig 2:**
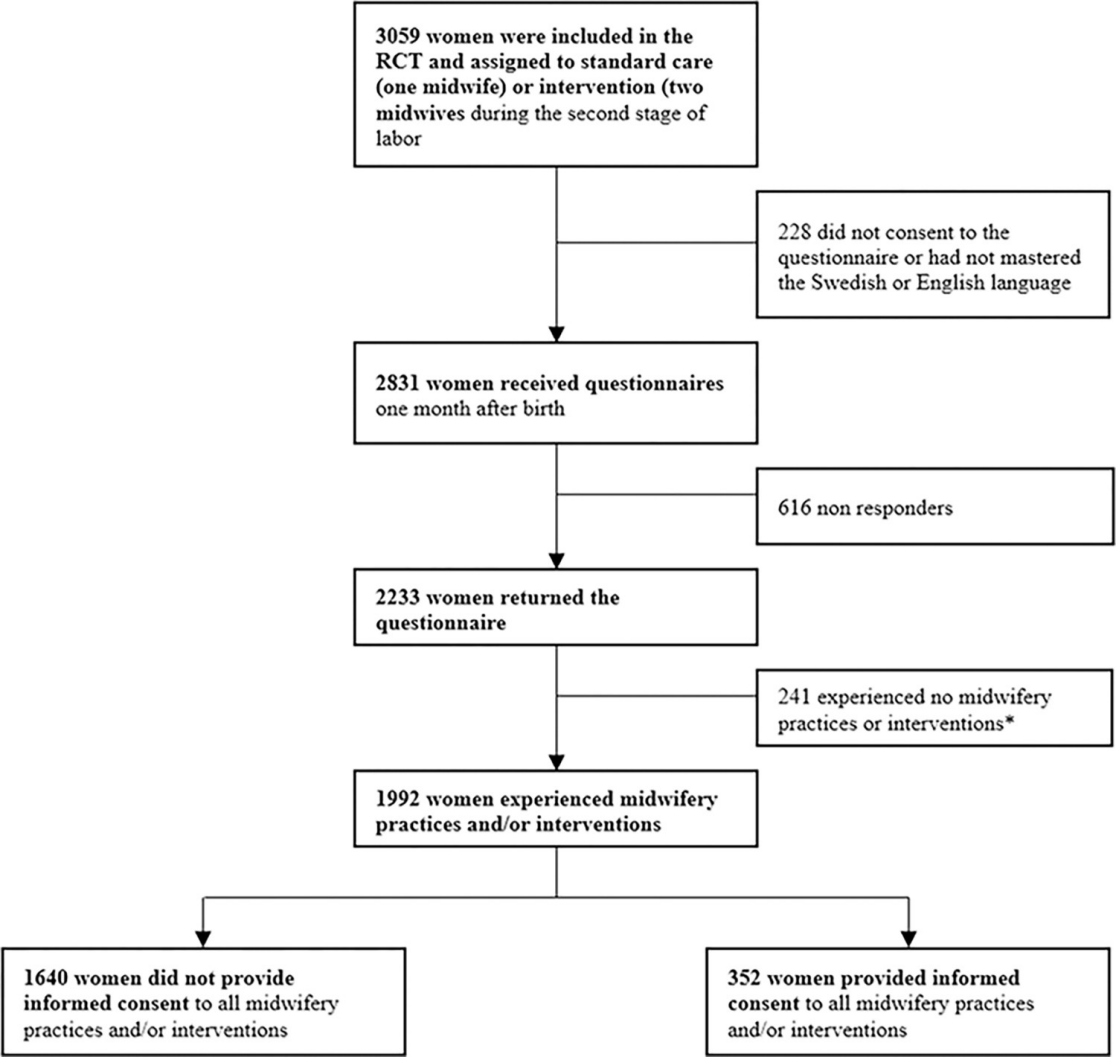
Flowchart over the participants in the study. * Women who responded that they had not experienced or could not recall any of the midwifery practices or interventions.

Out of the 2233 women who responded to the follow-up questionnaire, 87.6% responded within two months after their births and 11.5% responded between two and four months after their births resulting in a response rate of 78.9%. A total of 241 women (10.8%) experienced no midwifery practices or interventions during the second stage and were therefore not included in the analyses. Of the remaining 1992 women who recalled that the midwife had performed at least one midwifery practice or intervention, only a minority of 352 women (17.6%), had provided informed consent to all midwifery practices or interventions they had received (Fig 2). Provided informed consent refers to a combined variable including receiving information about a procedure and being given the option to decline it.

### Background and labor and birth characteristics

There were no differences between the groups regarding parity, age, BMI, marital status or ethnicity (Table 1). However, women who reported that they had provided informed consent were less likely to have a university education (65.3% *vs* 74.8%; p<0.001) (Table 1). A majority of the women had their labors augmented with synthetic oxytocin (69.4%) and had epidural analgesia as pain relief (62.4%) (Table 2). Furthermore, women who provided informed consent were less likely to give birth in lithotomy or recumbent position (29% *vs* 36%, p = 0.01) (Table 2).

**Table 1:** Background characteristics.

	Informed consent (N = 352)	No informed consent (N = 1640)	p-value
Parity			
Nulliparous	329 (93.5)	1531 (93.4)	0.94
VBAC	23 (6.5)	109 (6.6)	
Maternal age (mean, SD)	29.97 (4.23)	30.17 (4.23)	0.42
Maternal age group (years)			
<25	30 (8.5)	138 (8.4)	0.95
25–35	280 (79.5)	1315 (80.2)	0.79
>35	42 (11.9)	187 (11.4)	0.78
BMI groups			
<18.5	6 (1.7)	37 (2.3)	0.51
18.5–24.9	210 (59.7)	970 (59.1)	0.96
25–30.0	83 (23.6)	364 (22.2)	0.61
>30.0	38 (10.8)	189 (11.5)	0.67
Missing data	15 (4.3)	80 (4.9)	
Level of education			
Elementary school	7 (2.0)	37 (2.3)	0.76
Upper secondary school	102 (29.0)	335 (20.4)	<0.001
University†	230 (65.3)	1227 (74.8)	<0.001
Other	11 (3.1)	32 (2.0)	0.17
Missing data	2 (0.6)	9 (0.5)	
Marital status			
Living with a partner	320 (90.9)	1496 (91.2)	0.93
Not living with a partner or other	16 (4.6)	73 (4.5)	
Missing data	16 (4.6)	71 (4.3)	
Ethnicity			
Nordic	261 (74.1)	1249 (76.2)	0.53
European	39 (11.1)	155 (9.5)	0.33
African	7 (2.0)	24 (1.5)	0.46
Middle Eastern	18 (5.1)	92 (5.6)	0.73
South American	8 (2.3)	24 (1.5)	0.27
Asian	15 (4.3)	87 (5.3)	0.43
Missing data	4 (1.1)	9 (0.5)	
History of mental health issues before pregnancy	71 (20.2)	355 (21.6)	0.55
Missing data	2 (0.6)	7 (0.4)	
Fear of birth during pregnancy	72 (20.5)	345 (21.0)	0.80
Missing data	3 (0.9)	15 (0.9)	
Swedish as native language	256 (72.7)	1228 (74.9)	0.43
Missing data	3 (0.9)	11 (0.7)	

Data are n (%). VBAC = vaginal birth after caesarean section. BMI = body mass index. Comparisons between groups were calculated using students *t* test (continuous variables) and χ2 test (dichotomous variables).

*Total study population

^†^3–5 years

**Table 2:** Labor and birth characteristics.

	Informed consent (N = 352)	No informed consent (N = 1640)	p-value
Onset of labor			
Spontaneous	262 (74.4)	1209 (73.7)	0.78
Induction	90 (25.6)	431 (26.3)	
Epidural analgesia/spinal	231 (65.6)	1023 (62.4)	0.25
Birth position			
Lateral	134 (38.1)	586 (35.7)	0.46
Lithotomy/recumbent	102 (29.0)	590 (36.0)	0.01
Sitting	72 (20.5)	291 (17.7)	0.26
Kneeling/standing	18 (5.1)	80 (4.9)	0.87
Birth chair/squatting	13 (3.7)	50 (3.0)	0.55
All four	12 (3.4)	27 (1.6)	0.03
Missing data	1 (0.3)	16 (1.0)	
Augmentation with synthetic oxytocin	234 (66.5)	1167 (71.2)	0.08
Second stage of labor—minutes (median, IQR)	106 (60.0–176.0)	107 (62.0–169.5)	0.77
Missing data	1 (0.3)	3 (0.2)	
Active second stage—minutes (median, IQR)	35.0 (23.0–50.0)	35.0 (24.0–52.0)	0.66
Missing data	10 (2.8)	41 (2.5)	
Post partum heammorhage >500 ml	112 (31.8)	515 (31.4)	0.94
SPT	15 (4.3)	74 (4.5)	0.84
Birth weight (mean, SD)	3510 (421)	3517 (425)	0.48

Data are n (%). SPT = Severe perineal trauma. Comparisons between groups were calculated using Chi square test (dichotomous variables) and Mann Whitney U test (continuous variables).

### Midwifery practices and interventions during the second stage of labor

The rate of women experiencing the use of interventions and midwifery practices ranged from 5.3 to 60.8%. The use of warm compresses was the most commonly experienced midwifery practice (60.8%), whereas fundal pressure was the intervention least experienced (5.3%) (Table 3). The midwifery practice most commonly recalled and provided informed consent to was perineal massage (48.4%), whereas the lowest rate of informed consent was provided for episiotomy (20.1%) (Table 3). Among the midwifery practices, the rate of women reporting that they could not recall experiencing them, varied from 23.6% for warm compresses to 52.6% for manual perineal protection. The corresponding rate for the interventions were 9.7% for episiotomy, followed by approximately one in four women for fundal pressure, intermittent catheterization, and vaginal examinations (Table 3).

**Table 3:** Informed consent to midwifery practices and interventions during the second stage of labor.

	Total study population* N = 2233	I received information about the procedure (Strongly agree)	I was given the option to decline the procedure (Strongly agree)	Combined variable Informed consent^†^
**Midwifery practices**				
Warm compresses				
Yes	1358 (60.8)	601 (46.4)	777 (60.3)	491 (38.0)
No	330 (14.8)			
Cannot recall	527 (23.6)			
Missing data	18 (0.8)			
Manual perineal protection				
Yes	945 (42.3)	457 (50.4)	404 (44.7)	316 (34.8)
No	101 (4.5)			
Cannot recall	1175 (52.6)			
Missing data	12 (0.5)			
Perineal massage				
Yes	220 (9.9)	129 (60.0)	128 (60.1)	104 (48.4)
No	904 (40.5)			
Cannot recall	1098 (49.2)			
Missing data	11 (0.5)			
**Interventions**				
Vaginal examination				
Yes	960 (43.0)	571 (61.0)	399 (43.4)	330 (35.5)
No	247 (11.1)			
Cannot recall	1020 (45.7)			
Missing data	6 (0.3)			
Levator pressure				
Yes	355 (15.9)	210 (60.5)	156 (45.7)	129 (37.6)
No	1296 (58.0)			
Cannot recall	567 (25.4)			
Missing data	15 (0.7)			
Intermittent catheterisation				
Yes	788 (35.3)	538 (69.2)	294 (38.5)	252 (33.0)
No	905 (40.5)			
Cannot recall	508 (22.7)			
Missing data	32 (1.4)			
Fundal pressure				
Yes	119 (5.3)	53 (46.5)	31 (28.2)	24 (21.4)
No	1544 (69.1)			
Cannot recall	564 (25.3)			
Missing data	6 (0.3)			
Episiotomy				
Yes	160 (7.2)	82 (52.9)	33 (21.7)	31 (20.1)
No	1843 (82.5)			
Cannot recall	216 (9.7)			
Missing data	14 (0.6)			

Data are n (%)

*All women responding to the questionnaire including the 1992 who recalled and received at least one practice and/or intervention, and the 241 women who did not recall any practice or intervention.

^†^The combined variable *Informed consent* corresponds to the answers “Strongly agree” on the statements *I received information about the procedure*, and *I was given the option to decline the procedure*

Women who could recall whether that they had experienced vaginal examinations during the second stage had a significantly longer second stage (median 111 minutes, IQR 61–174) than women who could not recall (median 95 minutes, 95% CI 56–160; p-value <0.001).

All midwifery practices and interventions except fundal pressure were experienced as significantly more positive when informed consent had been provided (Table 4). Vaginal examinations (aOR 1.58; 95% CI 1.19–2.10) and intermittent catheterization of the bladder (aOR 1.48; 95% CI 1.08–2.03) were experienced as significantly more unpleasant and/or painful in the no informed consent group, and were the most common interventions that women recalled. Furthermore, episiotomy (aOR 3.92; 95% CI 1.57–9.80) and perineal massage (aOR 3.61; 95% CI 1.32–9.90) were experienced as significantly more painful and/or unpleasant when informed consent was not provided (Table 5).

**Table 4:** Women’s experiences of midwifery practices and interventions as positive during the second stage of labor in relation to informed consent.

	The experience was positive			
	Strongly agree	Less than strongly agree	OR (95%CI)	aOR (95%CI)
**Midwifery practices**				
Warm compresses				
Informed consent	455 (40.5)	36 (21.6)	2.48 (1.68–3.65)	2.69 (1.80–4.01)*
No informed consent	669 (59.5)	131 (78.4)	1.0 Ref.	1.0 Ref.
Manual perineal protection				
Informed consent	284 (40.9)	31 (14.8)	3.99 (2.65–6.01)	4.01 (2.62–6.14)*
No informed consent	411 (59.1)	179 (85.2)	1.0 Ref.	1.0 Ref.
Perineal massage				
Informed consent	99 (53.2)	5 (17.2)	5.46 (2.00–14.93)	6.01 (2.14–16.86)*
No informed consent	87 (46.8)	24 (82.8)	1.0 Ref.	1.0 Ref.
**Interventions**				
Vaginal examinations				
Informed consent	263 (44.9)	65 (19.2)	3.43 (2.50–4.71)	3.43 (2.47–4.75)*
No informed consent	323 (55.1)	274 (80.8)	1.0 Ref.	1.0 Ref.
Levator pressure				
Informed consent	98 (43.8)	31 (26.3)	2.18 (1.34–3.55)	2.10 (1.27–3.47)*
No informed consent	126 (56.3)	87 (73.7)	1.0 Ref.	1.0 Ref.
Intermittent catheterisation of the bladder				
Informed consent	176 (41.7)	76 (22.5)	2.47 (1.79–3.40)	2.43 (1.76–3.37)*
No informed consent	246 (58.3)	262 (77.5)	1.0 Ref.	1.0 Ref.
Fundal pressure				
Informed consent	14 (30.4)	10 (15.2)	2.45 (0.98–6.15)	2.39 (0.93–6.12)^†^
No informed consent	32 (69.6)	56 (84.8)	1.0 Ref.	1.0 Ref.
Episiotomy				
Informed consent	20 (40.8)	11 (10.7)	5.77 (2.48–13.44)	6.97 (2.69–18.03) ^‡^
No informed consent	29 (59.2)	92 (89.3)	1.0 Ref.	1.0 Ref.

Data are n(%)

*Adjusted for “Swedish as native language”, “fear of birth”, “level of education” and “length of the second stage”

^†^Adjusted for “fear of birth” and “level of education”

^‡^Adjusted for “Swedish as native language”, “fear of birth” and “level of education”

**Table 5:** Women´s experiences of midwifery practices and interventions as painful and/or unpleasant during the second stage of labor in relation to informed consent.

	It was painful and/or unpleasant			
	Disagree	Strongly agree, mostly agree, agree in part	OR (95%CI)	aOR (95%CI)
**Midwifery practices**				
Warm compresses				
Informed consent	450 (38.3)	37 (37.4)	1.04 (0.68–1.59)	1.08 (0.69–1.67)*
No informed consent	725 (61.7)	62 (62.6)	1.0 Ref.	1.0 Ref.
Manual perineal protection				
Informed consent	237 (36.5)	78 (31.0)	1.28 (0.94–1.75)	1.29 (0.94–1.79)*
No informed consent	413 (63.5)	174 (69.0)	1.0 Ref.	1.0 Ref.
Perineal massage				
Informed consent	97 (51.6)	7 (26.9)	2.89 (1.16–7.21)	3.61 (1.32–9.90)*
No informed consent	91 (48.4)	19 (73.1)	1.0 Ref.	1.0 Ref.
**Interventions**				
Vaginal examinations				
Informed consent	174 (41.1)	154 (30.7)	1.58 (1.20–2.07)	1.58 (1.19–2.10)*
No informed consent	249 (58.9)	347 (69.3)	1.0 Ref.	1.0 Ref.
Levator pressure				
Informed consent	71 (37.0)	57 (38.0)	0.96 (0.62–1.49)	0.88 (0.55–1.42)*
No informed consent	121 (63.0)	93 (62.0)	1.0 Ref.	1.0 Ref.
Intermittent catheterization of the bladder				
Informed consent	146 (36.9)	103 (28.5)	1.46 (1.08–1.99)	1.48 (1.08–2.03)*
No informed consent	250 (63.1)	258 (71.5)	1.0 Ref.	1.0 Ref.
Fundal pressure				
Informed consent	7 (24.1)	16 (19.5)	1.31 (0.48–3.61)	1.14 (0.40–3.22) ^†^
No informed consent	22 (75.9)	66 (80.5)	1.0 Ref.	1.0 Ref.
Episiotomy				
Informed consent	16 (30.8)	15 (14.7)	2.58 (1.15–5.76)	3.92 (1.57–9.80) ^‡^
No informed consent	36 (69.2)	87 (85.3)	1.0 Ref.	1.0 Ref.

Data are n(%)

*Adjusted for “Swedish as native language”, “fear of birth”, “level of education” and “length of the second stage”

^†^Adjusted for “fear of birth” and “level of education”

^‡^Adjusted for “Swedish as native language”, “fear of birth” and “level of education”

**Fig 3:**
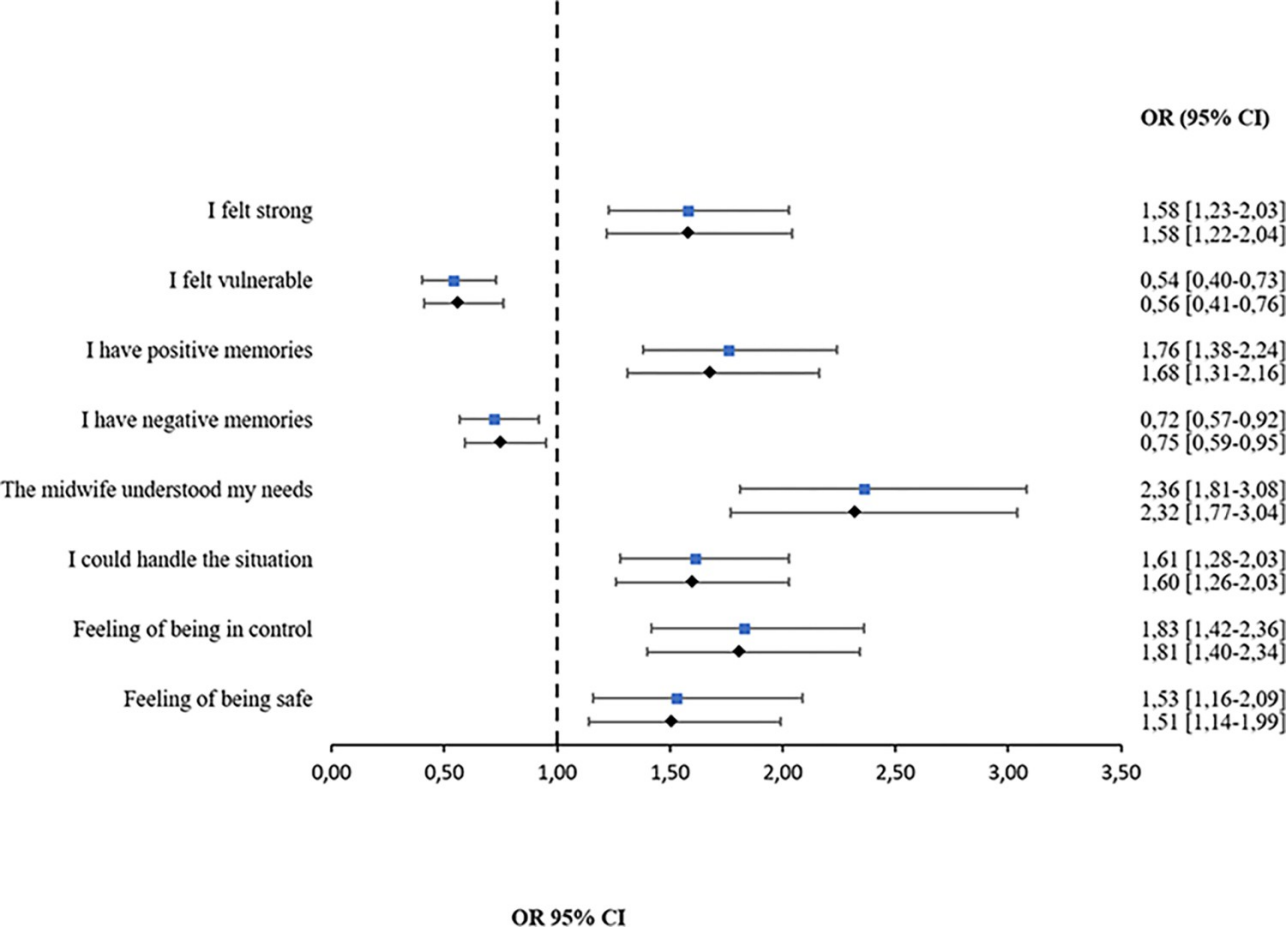
Forest plot showing the association between women’s experiences of the second stage of labor and having provided informed consent with crude and adjusted odds ratios.

Women in the informed consent group had significantly more positive experiences of the second stage of labor compared to women in the no informed consent group. This regards feelings of being strong (OR 1.58; 95% CI 1.22–2.04), handling the situation (aOR 1.60; 95% CI 1.26–2.03), having feelings of being in control (aOR 1.81; 95% CI 1.40–2.34) and of being safe (aOR 1.51, 95% CI 1.14–1.99). The strongest association was found between informed consent and experiencing that the midwife understood their needs (aOR 2.32; 95% CI 1.77–3.04). Women in the informed consent group also felt significantly less vulnerable (aOR 0.56; 95% CI 0.41–0.76) and had significantly fewer negative memories (aOR 0.75, 95% CI 0.59–0.95) (Table 6 and Fig 3).

**Table 6:** Women’s experiences during the second stage of labor in relation to informed consent.

	Informed consent (N = 352)	No informed consent (N = 1640)		
			OR (95% CI)	aOR (95%CI)*
I felt strong during the second stage of labor	118 (33.5)	396 (24.1)	1.58 (1.23–2.03)	1.58 (1.22–2.04)
I felt vulnerable during the second stage of labor	61 (17.3)	456 (27.8)	0.54 (0.40–0.73)	0.56 (0.41–0.76)
I have positive memories from the second stage of labor	128 (36.4)	404 (24.6)	1.76 (1.38–2.24)	1.68 (1.31–2.16)
I have negative memories from the second stage of labor	144 (40.9)	802 (48.9)	0.72 (0.57–0.92)	0.75 (0.59–0.95)
The midwife understood my needs during the second stage of labor	268 (76.1)	949 (57.9)	2.36 (1.81–3.08)	2.32 (1.77–3.04)
I could handle the situation during the second stage of labor	193 (54.8)	707 (43.1)	1.61 (1.28–2.03)	1.60 (1.26–2.03)
Feeling completely in control during the second stage of labor	116 (33.0)	349 (21.3)	1.83 (1.42–2.36)	1.81 (1.40–2.34)
Feeling totally safe during the second stage of labor	270 (76.7)	1128 (68.8)	1.53 (1.16–2.01)	1.51 (1.14–1.99)

Data are n(%)

*Adjusted for "Swedish as native language", "fear of birth", "level of education" and "length of the second stage"

## Discussion

In this study less than one in five women provided informed consent to all midwifery practices and interventions they experienced. The rates of provided informed consent were less than 50% for each of the practices and interventions. During the second stage of labor midwifery practices and interventions were experienced as significantly more positive when informed consent was provided. Women who perceived that they had provided informed consent to vaginal examinations, perineal massage, intermittent catheterization of the bladder and episiotomy experienced these interventions as less painful or unpleasant. Additionally, women who provided informed consent to all experienced practices and interventions felt more safe, strong and in control and less vulnerable during the second stage compared to women who had not provided informed consent.

The result from the present study shows that a minority of the women consented to midwifery practices and interventions during the second stage, ranging between 20% for episiotomy and 48% for perineal massage. These levels of informed consent are notably low. However, the lack of informed consent for episiotomy is in line with what is found in other studies [40–42]. In Sweden, the use of episiotomy is low, especially in spontaneous vaginal births, with a national rate of 6.9% for primiparous women in 2021 [43]. Given that Swedish midwives use episiotomy and fundal pressure restrictively [9], it might have been expected that they should be meticulous in providing information about the intervention and offering women the opportunity to decline, which was not the case in this study. Considering the low incidence of episiotomies, it is possible that they were performed in urgent situations where the midwife might consider informed consent to be of secondary importance. The lack of informed consent might thus reflect a previously described power imbalance between midwives and women [27], where midwives perform interventions they believe to be in the best interest of the woman. This was reported in an Australian study where midwives and physicians believed that a woman’s autonomy could be disregarded if they feared for the safety of the baby [44]. It has been suggested that care givers should prepare women antenatally for any unexpected and urgent decisions that may need to be taken during the birth [45]. However, the principle of autonomy includes the right to change/withdraw why informed consent will still be required during labor and birth [25]. Considering that both national and international guidelines [11, 46] state that obtaining consent from the woman should be mandatory before an episiotomy is performed, our results are concerning and further discussion among professions involved is warranted.

Vaginal examinations, perineal massage, intermittent catheterization of the bladder, and episiotomy were experienced as significantly less unpleasant or painful when informed consent was obtained. It is worth noting that all these interventions involve physical penetration of the genital area. Previous research has shown associations between such interventions and negative birth experiences, with the development of post-traumatic stress disorder in some women who had undergone the interventions [3, 16, 20, 47]. Our results align with the study by de Klerk et al., who found that 35% of the women reported negative experiences with vaginal examinations [19]. In addition, our findings contribute with evidence that negative experiences were further associated with the provision of informed consent. Since the results demonstrate that interventions involving physical penetration of the genital area can cause pain and lead to feelings of vulnerability and loss of control, midwives need to be particularly careful in providing women with information regarding the reason for the intervention, and ensure that women are given the opportunity to decline.

The findings from the present study are in line with previous research showing that involvement in decision-making, as well as having supportive relationships are factors related to positive experiences and feelings of being safe for women giving birth [48–50]. The strongest association observed in this study was found between informed consent and women’s experiences of the midwife understanding their needs during the second stage of labor. While respecting women’s right to informed consent, it is also important to recognize that women’s needs during childbirth are highly individual. Women may have different perspectives on the amount of information they wish to receive and the extent to which they want to be involved in decision-making processes [51, 52]. To be understood by the midwife may not only include providing informed consent, but also that the care giver is perceptive and compassionate so that individualized support is received.

A significant number of the women in this study were unable to recall experiences of specific practices and interventions. Considering the findings of Bossano et al (2017), which indicate that women generally have strong recollections of their birth experiences [53], it could be argued that the absence of memory regarding exposure to certain practices or interventions may not have a significant impact on women’s birth experiences. The intensified labor during the late second stage [54] can be experienced as overwhelming [2]. Women have described that they during this stage tend to withdraw from the outer world and focus on the physical task and how to handle increasing levels of pain [54]. This could potentially explain why women who had a shorter, and thus more intense second stage of labor, did not recall the use of vaginal examinations to the same degree as women who were able to recall their experiences.

Interestingly, we found no differences in the provision of informed consent among women with fear of birth or a history of mental health issues in this study, although it is known that these groups are at risk of having a negative birth experience [55, 56]. Even if our results do not provide evidence that informed consent is particularly important for these groups, there is still reason to believe that this is an important issue requiring further research. Furthermore, significantly less women with a university education had given their informed consent to midwifery practices and interventions, which may be attributed to the fact that that women with higher educational levels possess more information, have higher expectations, and therefore tend to be more critical. Higher educational attainment has previously been associated with negative experiences [55], but the opposite has also been reported [28]. This needs to be taken into consideration when interpreting the results.

### Strengths and limitations

The major strength of this study is its novel focus on informed consent during the second stage of labor and that it includes a variety of midwifery practices and interventions to study the association between informed consent and women’s experiences of the second stage. Furthermore, the study sample was collected prospectively, and the response rate was high. Using the response option “cannot recall” adds important information that some women did not remember the use of midwifery practices and interventions during this stage.

The study has some limitations that need to be considered. Firstly, the dichotomization of the Likert scales simplifies nuances of individual experiences, which can result in a loss of information about variations. Secondly, to increase the possibility of capturing the concept of informed consent, asking the women more specifically about informed consent should have been considered. The variable used in this study was created as a proxy for informed consent from two statements and women did not directly respond to a question whether they perceived that they had provided informed consent or not—something which needs to be considered when interpreting the results. Asking women further questions about informed consent i.e. how informed consent took place (verbally, non-verbally or by other means) and whether they declined the interventions being proposed could have increased the understanding of women’s preferences regarding information and having their choices respected during the second stage of labor. However, being provided with information, and having the option to decline an intervention are crucial aspects of informed consent according to Swedish law [26], and we designed the items to capture these parts of informed consent. Although the items in the questionnaire were tested for face validation, the psychometric properties have not been tested, consequently, values for internal consistency cannot be presented. Furthermore, the use of single items is a limitation. Since the experience of the second stage of labor has been shown to be complex and multidimensional [2] the use of single items might not fully capture this complexity, unlike if a scale consisting of multiple items measuring the various aspects of this experience were used. Validated scales regarding women’s experiences of the second stage and informed consent are therefore needed.

## Conclusion

The findings from this study indicate that informed consent during the second stage is associated with feelings of safety and of being in control. When women provided informed consent, they reported more positive experiences with the use of midwifery practices and interventions and less discomfort or pain from interventions involving physical penetration of the genital area. With less than one in five women reporting informed consent to all practices and interventions performed by midwives, the result emphasizes the need for further action to enhance midwives’ knowledge and motivation in obtaining informed consent prior to performance of interventions. To improve women’s experiences of the second stage of labor, individualized and optimized processes for obtaining informed consent are necessary. Future research should explore barriers to obtainment of informed consent and identify strategies to address these barriers.

## Supporting information

S1 Appendix Items measuring women’s experiences of the second stage of labour in the Oneplus follow-up questionnaire. (PDF)
