# Supplementary material for: Informed consent to midwifery practices and interventions during the second stage of labor—An observational study within the Oneplus trial
Source: PLoS One. 2024 Jun 12;19(6):e0304418. doi: 10.1371/journal.pone.0304418 (PMC11168622; doi:10.1371/journal.pone.0304418)
Supplement: S1 Appendix — (PDF) [file pone.0304418.s001.pdf]

S1 Appendix. Items measuring women's experiences of the second stage of labour in the Oneplus follow-up questionnaire

**Items rated on a 4-point Likert scale**
I felt strong during the second stage of labour
I could handle the situation during the second stage of labour
I was tired during the second stage of labour
I have positive memories from the second stage of labour
I have negative memories from the second stage of labour
I felt vulnerable during the second stage of labour
I was afraid during the second stage of labour
I was concerned about my child´s health during the second stage of labour
The midwife understood my needs during the second stage of labour
I felt included in decision about birth position
**Items rated on a 7-point Likert scale**
How much of a feeling of being in control did you experience during the second stage of labour?<br>*not in control (1) – completely in control (7)*
During the second stage of labour I felt:<br>*no pain at all (1) – worst imaginable pain (7)*
I experienced the pain as:<br>*very negative (1) – very positive (7)*
How did you experience the length of the second stage of labour?<br>*drawn out (1) – fast (7)*
When you look back on the birth now, how safe did you feel during the second stage of labour?<br>*very unsafe (1) – totally safe (7)*

The 4-point Likert scales range from 1 (Strongly agree) to 4 (Disagree).
